# Editorial: Understanding leukemia biology using genome editing techniques

**DOI:** 10.3389/fonc.2023.1323584

**Published:** 2023-11-07

**Authors:** Silvia Jimenez-Morales, Kaushik Banerjee, Nirmalya Saha, Amrita Basu, Kathy L. McGraw

**Affiliations:** ^1^ Laboratorio de Innovación y Medicina de Precisión, Núcleo “A”, Instituto Nacional de Medicina Genómica, Mexico City, Mexico; ^2^ Department of Neurosurgery, School of Medicine, Michigan Medicine, University of Michigan, Ann Arbor, MI, United States; ^3^ Department of Pathology, Michigan Medicine, University of Michigan, Ann Arbor, MI, United States; ^4^ Department of Pathology and Laboratory Medicine, Diagnostic Immunology Lab, Nationwide Children’s Hospital, Columbus, OH, United States; ^5^ Laboratory of Receptor Biology and Gene Expression (LRBGE), Center for Cancer Research (CCR), National Cancer Institute (NCI), National Institutes of Health (NIH), Bethesda, MD, United States

**Keywords:** leukemia, myeloid leukemia, lymphoid leukemia, CD25, PD-1, lysosomal activation, mitochondrial gene expression

Leukemia represents a life adjusting, high mortality inducing health problem, accounting for approximately 2.5% of all new cancer diagnoses and 3.1% of cancer-related mortality worldwide, which is estimated to increase future years (1, 2). The term Leukemia comes from the Greek word ‘leukos’, meaning white, and ‘haima’, meaning blood and includes a number of hematologic neoplasia that show high heterogeneity in their clinical course and phenotype including vast differences in immunophenotypes, associated cytogenetics, and molecular features (3). Based on the integration of morphologic, clinical, and genomic data, the “WHO Classification of Tumours of Haematopoietic and Lymphoid Tissues (2016)” identifies 12 major International Consensus Classification categories of myeloid neoplasms and acute leukemias, which can be broadly classified into myeloid or lymphoid (4). The causes of leukemia are not completely understood, but a plethora of evidences reveal that these malignancies emerge from acquired mutations during fetal or postnatal hematopoiesis, which alter cell differentiation, leading to dysregulated hematopoiesis and in some cases uncontrolled proliferation of transformed cells (5). It is well recognized that the number and type of mutations can direct the course of disease, amplifies leukemia complexity, and can determines patient prognosis. In this issue, five original research articles present various aspects of cutting-edge, sophisticated research aimed to address the scope and prospects of gene editing in myeloid and lymphoid leukemia.

Towards the goal of personalized medicine, enormous efforts are underway to increase overall survival in leukemia patients by developing new targeted treatments with maximum leukemic cell selectivity and minimum toxicity. Using CRISPR/Cas9 and shRNA gene editing, Shah et al. explored potential lysosomal vulnerabilities as a targeted therapeutic approach for myeloid leukemia treatment. For this purpose, leukemic cell lines, blasts isolated from pediatric AML patients, and expanded CD34+-hematopoietic stem cells derived from donors were used. The authors observed larger lysosomal compartments, with greater lysosome activity, and an increased lysosomal membrane sensitivity in leukemic cells compared to donor cells. Importantly the authors demonstrated that the combination of mefloquine plus mTOR inhibitors selectively increases leukemic cell death through disruption of lysosomal compartments. These data suggest that targeting the lysosome, particularly in chemotherapy-resistant cells, may serve as an effective therapeutic strategy not only for leukemia but potentially for other types of malignancies as well.

It is as well-known that γδ T cells are potent activators of anti-tumor cytotoxicity and have important roles in initiating and establishing immune responses, however differences in γδ T cell populations within leukemic patients may have clinical indications. Zheng et al. analyzed γδ T cell populations and correlated with immune- checkpoint mediated T cell immune dysfunction and *PD-1* and *FOXP3* gene expression, and prognosis in acute myeloid leukemia (AML) patients. To this end, RNA-seq data were retrieved from the TCGA dataset and γδ T cells were sorted from peripheral blood of *de novo* AML adults or healthy subjects by flow cytometry. The data demonstrated higher PD-1 expression levels in AML cases compared with healthy individuals and a correlation of elevated PD-1+Foxp3+ γδ T cells with poor clinical outcome. These data support future investigations of γδ T cells in AML to understand population differences and functional consequences of differentially expressed genes within these subsets. Ultimately, these data suggest that γδ T cell may also be a novel therapeutic targeting strategy.

Based on the altered expression of CD25/IL2RA in AML and its association with poor survival; Pousse et al. dissected the immune cell composition of peripheral blood (PB) and bone marrow (BM), and the phenotype of CD25+ blasts from AML cases compared to controls. Significant heterogeneity in the myeloid compartment, a higher proportion of Tregs, and higher expression of CD25 in BM Tregs compared to B or T cells were observed in AML cases *versus* controls. *Ex vivo* treatment of leukemic cell isolated from patients revealed that monoclonal antibody targeting of CD25 is highly specific for killing CD25+ AML cells and Tregs. These data underscore the notion that deep characterization of cell populations is a promising area of further investigation to develop new therapeutic approaches in AML.

Chaudhary et al. evaluated the gene expression profile of mitochondrial-related genes for identification of prognostic biomarkers in pediatric AML. By integrating data from mtDNA copy number and whole transcriptome sequencing, the authors identified mitochondria-related differentially expressed genes (DEGs) and associated with clinical characteristics. Increased mtDNA copy number was significantly associated with poorer event free survival and *SDHC*, *CLIC1*, and *SLC25A29* expression levels were found to be predictive of worse event free survival and overall survival. *SLC25A29* results were validated in a TCGA adult AML dataset. A prognostic gene signature including *SDHC*, *CLIC1*, and *SLC25A29* was found to be independently predictive of survival. Moreover, *CLIC1* emerges as a potential therapeutic target in AML.

Based on the hypothesis that Herpesvirus entry mediator (HVEM) expression on hematopoietic tumor cells can modulate the anti-tumor response, del Rio et al. assessed the role of HVEM in tumor survival through adoptive transfer of HVEM WT or HVEM KO obtained by CRISPR/Cas9 gene editing in A20 leukemia cells into mouse models. The authors found that the absence of HVEM expression reduces tumor progression and colonization of leukemia cells to hematopoietic extramedullary organs by increasing NK and NKT cells and showed that PD-1- T cells were the most relevant player for inhibiting tumor engraftment. This work demonstrates that inhibiting the expression of HVEM is a potential mechanism to reduce leukemia progression and potentially a novel therapeutic approach.

In conclusion, leukemia represents a group of heterogeneic, hematologic malignancies with high genomic complexity and outcome diversity. This Research Topic highlights advances in the understanding of critical aspects of the biology and treatment of leukemia, to facilitate development of effective therapeutic strategies for improved patient outcome. As Guest Editors for this Research Topic, we hope that the readers enjoy reviewing these papers.

## Author contributions

SJ-M: Conceptualization, Investigation, Writing – original draft, Writing – review & editing. KB: Writing – review & editing. NS: Writing – review & editing. AB: Writing – review & editing. KM: Writing – review & editing.
